# Tailoring PLA/Gelatin Film Properties for Food Packaging Using Deep Eutectic Solvents

**DOI:** 10.3390/molecules31010039

**Published:** 2025-12-22

**Authors:** M. Cidália R. Castro, João Pereira, Mara Pires André, Pedro Pereira, Vasco Cruz, Pedro Veiga Rodrigues, Ana Vera Machado

**Affiliations:** Institute for Polymers and Composites (IPC), Department of Polymer Engineering, University of Minho, 4804-533 Guimarães, Portugal; cidaliacastro@dep.uminho.pt (M.C.R.C.); b13560@dep.uminho.pt (J.P.); pg55776@alunos.uminho.pt (M.P.A.); pg55778@alunos.uminho.pt (P.P.); vasco.cruz@dep.uminho.pt (V.C.); pedro.rodrigues@dep.uminho.pt (P.V.R.)

**Keywords:** fish residues, deep eutectic solvent (DES), sustainable polymer design, biodegradable packaging

## Abstract

This work investigates the modification of poly(lactic acid) (PLA) film properties for food packaging applications through the incorporation of modified gelatin (Gel-mod) and a choline chloride/glycerol deep eutectic solvent (DES). PLA/Gel-mod/DES materials were melt-processed and evaluated with respect to structure, morphology, thermal and mechanical behavior, processability, wettability, barrier performance, and compostability. Two incorporation routes were investigated for adding Gel-mod into the PLA matrix: direct incorporation and masterbatch preparation. FTIR and SEM analyses confirmed improved interfacial interactions and more homogeneous dispersion when Gel-mod was directly incorporated, compared with the masterbatch route. DES acted as an effective plasticizer and nucleating agent, reducing Tg, increasing crystallinity, and enhancing processability while maintaining thermal stability. Mechanical properties decreased relative to neat PLA, primarily due to increased crystallinity and chain scission. PLA_4Gel-mod demonstrated a more balanced performance, with higher elongation at break and improved processability than the other formulations, likely due to its single processing cycle, which minimized PLA degradation. Increased hydrophilicity led to higher water vapor transmission rates, correlating with accelerated biodegradation. Overall, the synergistic incorporation of DES and gelatin provides a viable strategy to tailor PLA properties, enabling the development of compostable packaging films suitable for sustainable food contact applications.

## 1. Introduction

The global transition toward sustainable and circular material systems is accelerating the replacement of petroleum-based plastics with biodegradable polymers obtained from renewable resources [1]. Poly(lactic acid) (PLA) is one of the leading candidates for food packaging applications due to its high mechanical strength, excellent transparency, and favorable barrier performance [2]. However, its intrinsic brittleness, limited thermal stability, and poor toughness limit its use in demanding packaging environments, making material modification a pressing scientific and industrial challenge [3].

Simultaneously, the valorization of food-industry by-products has emerged as a promising route to reduce waste streams and environmental impact while enabling the development of bio-based packaging materials with enhanced functionality [4]. Fish gelatin is particularly attractive because of the substantial volumes of residues generated during fish processing (where discard fractions can reach 50–70%), offering a sustainable and underexploited precursor for polymer formulations [5]. Gelatin provides excellent film-forming ability, high extensibility, optical clarity, and abundant amino and hydroxyl groups capable of interacting with PLA [6,7]. Nonetheless, PLA/gelatin blends typically suffer from significant phase separation and weak interfacial adhesion due to their chemical incompatibility, resulting in impaired mechanical integrity and reduced barrier properties [8,9,10]. Effective compatibilization strategies are therefore essential to enhance the potential of this system.

Deep eutectic solvents (DES), formed from a hydrogen-bond acceptor such as choline chloride and a hydrogen-bond donor such as glycerol, have recently emerged as a new generation of green compatibilizers and plasticizers. They offer advantages including low toxicity, biodegradability, negligible volatility, and simple synthesis without purification. Their extensive hydrogen-bonding networks enable strong molecular interactions with both PLA carboxyl groups and gelatin amino/hydroxyl functionalities, promoting homogeneous dispersion, enhanced interfacial adhesion, and improved rheological and mechanical behavior [11,12,13]. Moreover, combining DES with reactive crosslinkers, such as 2-hydroxyethyl methacrylate (HEMA), may further reinforce interfacial interactions and enable tailored performance for advanced food packaging applications [14]. The combination of PLA, gelatin, DES, and crosslinkers offers an opportunity to design high-performance films suitable for food packaging.

Despite growing interest in DES-modified biopolymers, no studies to date have reported melt-extrusion processing of PLA/gelatin materials compatibilized with DES, representing a notable gap in the field. Moreover, the synergistic effect of DES and HEMA in improving miscibility and end-use performance remains largely unexplored.

This study demonstrates the successful incorporation of gelatin-mod into PLA using a deep eutectic solvent (choline chloride/glycerol) and HEMA. Films were produced via compression molding and comprehensively evaluated to establish structure–property relationships and assess their potential for sustainable food packaging. By combining renewable raw materials, green compatibilization chemistry, and industrially relevant processing, this work contributes to replacing conventional plastics with next-generation biodegradable packaging solutions.

## 2. Materials and Methods

### 2.1. Reagents

PLA (LX175) was obtained from TotalEnergies (Gorinchem, the Netherlands), while gelatin (Gel) from cold-water fish skin and choline chloride, ≥98.5 (ChCl), were purchased from Sigma-Aldrich (Darmstadt, Germany). Glycerol (Gly) and 2-Hydroxyethyl methacrylate ≥ 99% (HEMA) were supplied by Alfa Aesar (Karlsruhe, Germany).

### 2.2. Methods

To determine the maximum feasible gelatin content in PLA and assess its processability during extrusion conditions, preliminary trials were conducted using several gelatin/DES/PLA ratios, and the resulting outcomes were used to establish the compositions investigated in this study (Table 1).

First, choline chloride (ChCl) and PLA were dried in a vacuum oven at 70 °C for 24 h prior to use. The DES homogeneous solution was prepared by mixing ChCl and Gly (ChCl:Gly) using a 1:2 molar ratio, at 90 °C under magnetic stirring. This ratio was selected based on their thermal stability reported by Delgado-Mellado et al. [13]. Then, to prepare the solution of modified gelatin (Gel-mod) as listed in Table 1, HEMA was added to the pre-prepared DES mixture. After homogenization, fish gelatin was incorporated and stirred for 2 hrs to complete dissolution.

Prior to the extrusion process, a PLA/Gel-mod masterbatch (20% *w*/*w* of Gel-mod) was prepared in a Haake Rheomix batch mixer (Roller Rotors R600, 50 cm^3^, Karlsruhe, Germany), equipped with counter-rotating rotors, at 170 °C and 80 rpm for 5 min. Then, pristine PLA was mixed with the prepared masterbatch to produce the compositions described in Table 2. After drying at 80 °C overnight in a vacuum oven, neat PLA and the mixtures PLA/Gel-mod were processed in a Leistritz LSM co-rotating twin-screw extruder (L/D of 29, with two mixing zones of 4KB30° and 4KB-30°, Nuremberg, Germany) with a temperature profile of 175 °C (feeding zone)/175 °C/175 °C/175 °C/170 °C/170 °C/160 °C (die) and a screw speed of 100 rpm/3 kg.h^−1^. The compositions PLA_1.5DES and PLA_3DES were prepared to assess the influence of DES and HEMA on the PLA matrix, using the same ratio of DES-HEMA/PLA as PLA_1.5MB and PLA_3MB. DES and HEMA were directly added to PLA during extrusion, using a syringe pump. Due to the high viscosity of Gel-mod, to prepare PLA_4Gel-mod (4% of modified gelatin), the same extruder preparation was used.

To evaluate the material’s properties, the extruded filament was first granulated, and then films were prepared by compression molding in a hot press (Moore H05012, Birmingham, UK) at 190 °C. About 10 g of material was placed between two plates without pressure, then 10 tons of pressure was applied for 2 min. After this time, a cooling cycle to room temperature (using water circulation) was applied.

**Figure 1:**
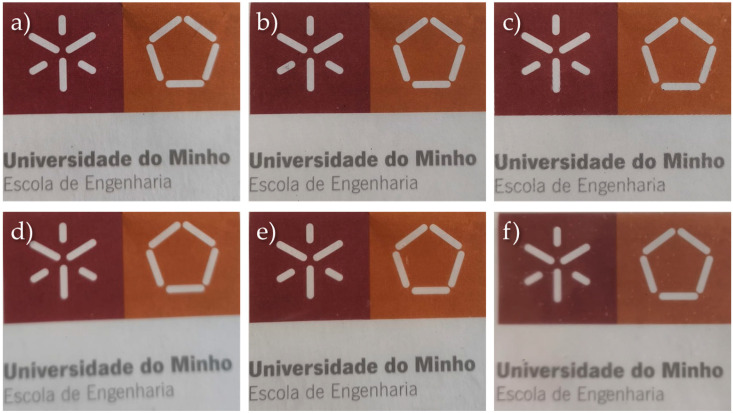
Photographs of the produced films: (**a**) PLA, (**b**) PLA_1.5DES, (**c**) PLA_3DES, (**d**) PLA_1.5MB, (**e**) PLA_3MB, and (**f**) PLA_4Gel-mod.

Figure 1 presents the resulting films. Overall, the samples exhibited a visual appearance comparable to neat PLA, regardless of the addition of DES or Gel-mod, and no perceptible macroscopic changes were observed with increasing Gel-mod content.

### 2.3. Films Thickness

Films’ thickness was measured using a hand-held digital film thickness gauge (Mitutoyo Absolute, No. 547-301, Kawasaki, Japan). At least five random points were measured per film, and the average value was reported.

### 2.4. Structural and Morphological Characterization

Fourier transform infrared spectroscopy (FTIR) was performed using a Jasco 4100 spectrometer (Tokyo, Japan) in transmittance mode, with 32 scans, 4 cm^−1^ resolution, and a spectral range from 4000 to 600 cm^−1^.

Morphological characterization of fractured specimens was performed using a NOVA 200 NanoSEM (FEI Company, Hillsboro, OR, USA). Films were previously fractured in liquid nitrogen and coated with a thin film (2 nm) of 150 Au-Pd (80–20 wt.%) in a high-resolution sputter coater (208HR Cressington Company, Watford, UK), coupled to an MTM-20 Cressington High Resolution Thickness Controller (Watford, UK).

### 2.5. Thermal Characterization

Thermogravimetric analysis (TGA) was performed in accordance with ISO 11358-1:2014 [15], using a Q500 instrument (TA Instruments, New Castle, DE, USA) from 40 to 400 °C under N_2_ atmosphere at 10 °C/min.

Differential scanning calorimetry (DSC) was conducted with a Netzsch 200 (Netzsch, Selb, Germany) under nitrogen, from 40 to 180 °C with a heating rate of 10 °C/min to evaluate the thermal transitions as follows ISO 11357-1:2016 [16]. An initial heating cycle was performed to remove the thermal history. The degree of crystallinity (Xc) of PLA was calculated using the following:

(1) $$Xc=\frac{{∆H}_{m}}{(1-\phi ){∆H}_{0}}\times 100,$$

where *ϕ* is the PLA weight fraction of the dispersed phase, ΔHm is the melting enthalpy (J/g) from the DSC curve, and ΔH_0_ is the heat of fusion for completely crystallized PLA (93.0 J/g) [17].

### 2.6. Melt Flow Index (MFI)

To assess the materials’ processability, the melt flow index was determined using Daventest equipment (Lenzing Instruments, Lenzing, Austria) at 190 °C under a 2.16 kg load, following ASTM D1238-04 standard [18]. At least three tests were performed for each composition.

### 2.7. Mechanical Properties

Tensile specimens (25 × 150 mm) were cut from the compressed molded films. Tensile tests were performed on a Zwick/Roell Z005 universal testing machine (Zwick/Roell, Ulm, Germany), at a 2 mm/min velocity and a grip separation of 100 mm, following ASTM D882-02 standard [19]. At least 5 specimens were tested, and the mean values were reported.

### 2.8. Contact Angle

Contact angle (CA) measurements were performed to assess surface hydrophobicity using a goniometer (Contact Angle System OCA 20, Dataphysics, Germany). Distilled water, 3 μL, was deposited on the film surface using a precision syringe, following the sessile drop method (ISO 19403-2) [20]. The first image of the droplet was captured at 0 s using an integrated video camera. At least five measurements were performed per film to calculate the CA mean value.

### 2.9. Water Vapor Transmission

The water vapor transmission rate (WVTR) was determined using the desiccant method described in ASTM E96/E96 M-05 [21]. The films were placed in a circular metallic test cup (surface diameter of 69.5 mm) filled with dried CaCl_2_ and sealed with paraffin wax to ensure that vapor transport occurred only through the film. The test cups were placed in a desiccator at constant temperature (21.02  ±  2.07 °C) and relative humidity (93.47  ±  4.75%) RH and weighed daily for one month. WVTR was calculated from the linear region of the curve.

### 2.10. Compostability

Compression-molded specimens (5 × 2 cm) were subjected to biodegradation testing in a compost environment, consisting of soil and activated sludge obtained from wastewater treatment facilities, kept at 40 °C under controlled relative humidity levels of about 50–55%. Aerobic conditions were ensured by positioning the samples vertically at depths of 4–6 cm, spaced at least 5 cm apart. After 10, 14, 18, and 21 days, the specimens were removed, washed, and air-dried at ambient temperature to a constant weight. Three replicates were tested for each film type, and the residual weight percentage was calculated.

### 2.11. Statistical Analysis

Data are reported as mean ± standard deviation. Statistical analysis was performed by one-way ANOVA, followed by Tukey’s post hoc test (*p* < 0.05) using SPSS software, version 22.0 (IBM Corp., Armonk, NY, USA).

## 3. Results and Discussion

### 3.1. Structural and Morphological Properties

#### 3.1.1. Fourier Transform Infrared Spectroscopy (FTIR)

Fourier transform infrared spectroscopy (FTIR) was used for the identification of structural modifications in the polymer systems and to confirm the chemical integrity of the components. Accordingly, FTIR analyses were performed to assess the following: (i) the influence of DES and HEMA as a crosslinker on gelatin structure during the modification process; (ii) the effect of Gel-mod when incorporated into the PLA matrix; and (iii) the preservation of functional groups after extrusion.

**Figure 2:**
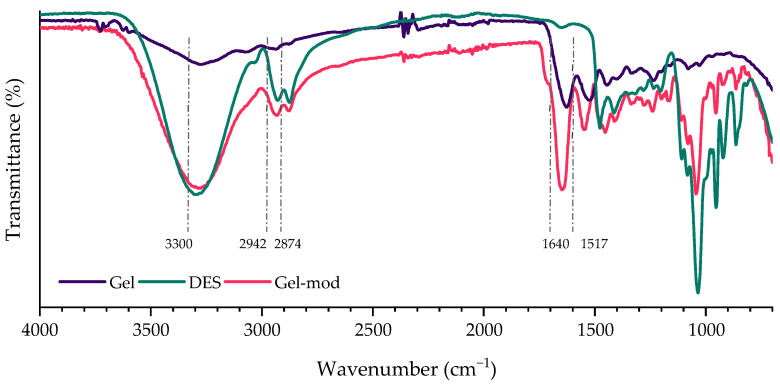
Spectra of neat gelatin, DES, and modified gelatin (Gel-mod).

The Gel-mod spectrum (Figure 2) shows the characteristic vibrational bands of fish gelatin, in agreement with those previously reported by Wu et al. [22], along with bands attributable to DES components. A broad absorption band around 3300 cm^−1^ corresponds to the stretching vibrations of N–H and O–H groups from the hydroxyl-rich gelatin and the DES. Bands between 2942 and 2874 cm^−1^ are associated with C–H (CH_2_) stretching of both constituents. The amide C=O and N–H bending bands of neat gelatin, located at 1640 and 1517 cm^−1^, respectively, undergo a slight blue shift to 1645 and 1546 cm^−1^ in the Gel-mod spectrum, suggesting the formation of ionic interactions between DES components and the carbonyl groups of gelatin [23].

**Figure 3:**
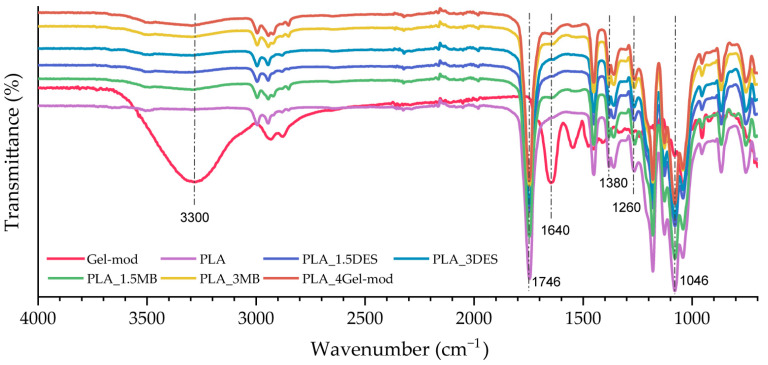
Spectra of modified gelatin (Gel-mod), PLA, and produced compositions (PLA_1.5DES, PLA_2DES, PLA_1.5MB, PLA_3MB, and PLA_4Gel-mod).

Regarding Figure 3, all prepared compositions exhibit the characteristic absorption bands associated with the PLA matrix [24]. The prominent peak at 1746 cm^−1^ corresponds to the stretching vibration of the carbonyl group (C=O) from both ester functionalities and terminal carboxylic acid groups. The bands located between 1380 and 1360 cm^−1^ are attributed to C–H stretching of methyl groups (CH_3_), while those observed in the 1260–1046 cm^−1^ region are assigned to C–O–C stretching typical of aliphatic ether linkages. In the compositions containing Gel-mod (PLA_1.5MB, PLA_3MB, and PLA_4Gel-mod), an additional broad band is observed between 3590 and 3110 cm^−1^, corresponding to O–H and N–H stretching vibrations, together with a peak at 1640 cm^−1^ associated with the C=O stretching of amide groups. These signals are more pronounced in the PLA_4Gel-mod composition due to its higher gelatin content. The results confirm that Gel-mod maintains its chemical integrity after extrusion and remains successfully incorporated into the PLA matrix, without signs of degradation or loss.

#### 3.1.2. Scanning Electron Microscopy

**Figure 4:**
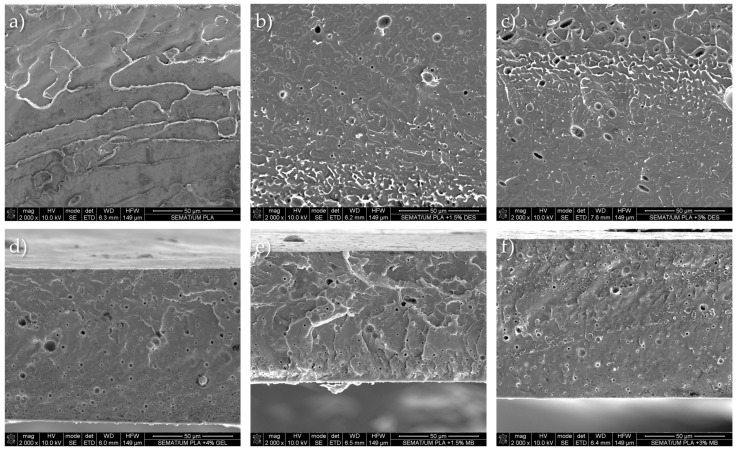
Cross-section SEM micrographs of (**a**) PLA, (**b**) PLA_1.5DES, (**c**) PLA_3DES, (**d**) PLA_4Gel-mod, (**e**) PLA_1.5MB, and (**f**) PLA_3MB films.

Scanning Electron Microscopy (SEM) was used to examine the fracture surface morphology and assess interfacial interactions between the PLA matrix, DES, and Gel-mod (Figure 4). The micrograph of neat PLA (Figure 4a) displays a relatively smooth fracture surface, characteristic of brittle failure, which is consistent with previous reports [17,25]. Localized regions of plastic deformation are also visible, suggesting that the film thickness enables partial yielding under plane-stress fracture conditions. Incorporation of gelatin through the masterbatch approach (Figure 4e,f) produces a noticeably different morphology, characterized by heterogeneous fracture surfaces and pronounced grooves, indicating increased brittleness compared with neat PLA. In the PLA_1.5MB and PLA_3MB formulations, the Gel-mod phase appears well-dispersed and embedded within the matrix, with PLA_3MB exhibiting a finer structural pattern. Debonded particles at the interface suggest partial compatibilization between Gel-mod and PLA, although interfacial failure is still detectable. In contrast, PLA_4Gel-mod formulation (Figure 4d), prepared via direct incorporation, displays fracture features more comparable to neat PLA, including regions of plastic deformation. The dispersed phase is considerably less apparent than in PLA_3MB, indicating improved interaction and interfacial adhesion between the protein-modified gelatin and the PLA matrix. This confirms that the direct incorporation route improves morphological homogeneity and minimizes degradation relative to the masterbatch approach [26].

### 3.2. Thermal Properties

**Figure 5:**
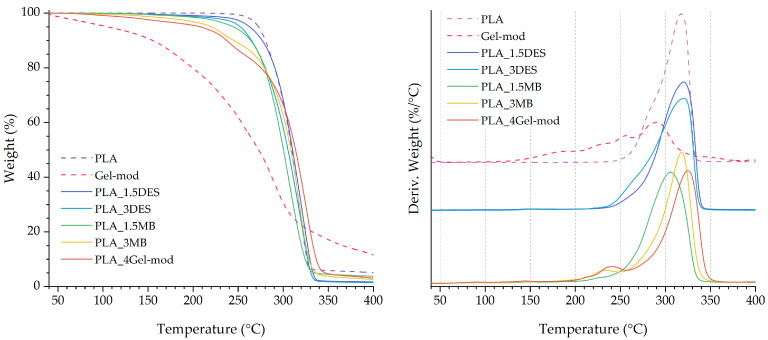
Thermograms of weight loss (**left**) and first derivative (**right**) of Gel-mod, PLA, and produced compositions.

**Figure 6:**
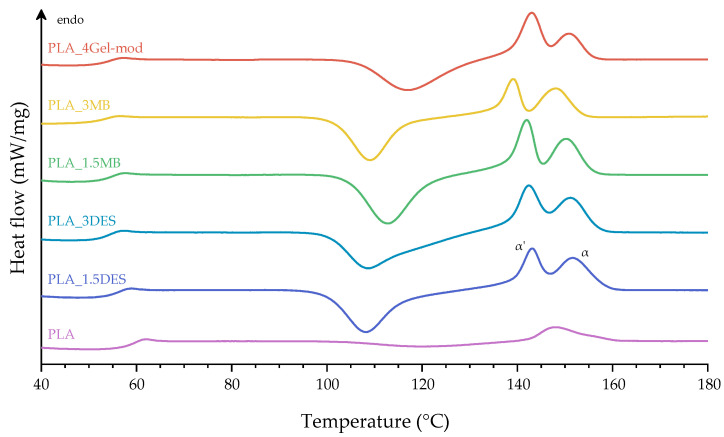
DSC thermograms of neat PLA and produced compositions (α′ and α indicates the less ordered and more ordered crystalline phases of PLA, respectively).

Thermal analysis was conducted to assess the influence of Gel-mod and DES on the thermal behavior of the PLA matrix (Figure 5 and Figure 6). The thermal transitions determined from the second heating cycle of the DSC measurements (glass transition temperature (Tg), cold crystallization temperature (Tc), melting temperature (Tm), melting enthalpy (ΔHm), and crystallinity degree (Xc)), as well as the thermal degradation peak temperature (Tpeak) obtained from TGA, are summarized in Table 3.

The thermogravimetric profile of Gel-mod exhibits multiple mass-loss events. The initial mass loss up to 150 °C is attributed to the release of free and bound water absorbed within the gelatin network. Subsequent degradation occurring between 200 and 260 °C corresponds to gelatin molecular decomposition, involving cleavage of amino acid chains and other organic constituents [19], accounting for approximately 30% of the total mass. A further mass-loss event around 290–300 °C is assigned to the thermal decomposition of the DES components, involving glycerol evaporation (≈290 °C) and choline chloride degradation (≈300 °C), in accordance with previously reported values, contributing to approximately 40% additional mass loss [13,27]. Considering the degradation profile of Gel-mod, some decrease in thermal stability was expected for the produced systems relative to neat PLA, which exhibits a degradation peak around 317 °C. The formulations incorporating Gel-mod displayed degradation peaks within the range of 305–325 °C, indicating that increasing Gel-mod content directly influences thermal degradation behavior and introduces an additional mass-loss event near 230 °C.

In contrast, the incorporation of Gel-mod into the PLA matrix results in two distinct mass-loss events, corresponding, respectively, to gelatin degradation (approximately 9–11% mass loss) and PLA thermal decomposition (approximately 3–11% mass loss). In the thermograms of PLA_1.5MB, PLA_3MB, and PLA_4Gel-mod, the first degradation peaks occur at approximately 225 °C, 230 °C, and 237 °C, with associated mass losses of 3, 9, and 12%, respectively. The primary degradation peaks for these formulations are observed at 306 °C, 318 °C, and 325 °C, respectively. These results indicate that increasing the percentage of Gel-mod enhances the overall composition’s thermal stability. This behavior may be attributed to strengthened polar interactions and hydrogen bonding between gelatin functional groups and the PLA matrix, which likely contribute to improved structural cohesion and resistance to thermal degradation.

DSC results (Figure 6 and Table 3) reveal the typical thermal transitions of neat PLA, characterized by a glass transition temperature (Tg) of approximately 58 °C, a cold crystallization peak (Tc) near 120 °C, and a broad double melting endotherm at 148 and 155 °C, corresponding to the coexistence of the α′ and α crystalline phases, respectively [28]. The incorporation of DES leads to a reduction in Tg, which indicates that they act as plasticizing agents within the PLA matrix. This reduction suggests that DES disrupts intermolecular interactions and enhances chain mobility, thereby decreasing the energy required for segmental relaxation [29]. Consistently, the lower Tc values observed for PLA_1.5DES and PLA_3DES reflect easier chain rearrangement and accelerated crystallization kinetics during heating. The plasticization effect promotes the development of more ordered and thermodynamically stable crystalline domains, as indicated by the increased intensity of the α-phase melting peak. Accordingly, the degree of crystallinity (Xc) increases substantially from 11% for neat PLA to values approaching 48% in the DES and Gel-mod-containing formulations. The highest crystallinity observed for PLA_3DES (≈48%) suggests that DES may additionally act as a nucleating agent, further accelerating crystal formation. Although the incorporation of Gel-mod does not significantly change Tg, it induces notable changes in the crystalline morphology of PLA, as reflected in the narrower melting endotherms. Moreover, the melting peak of PLA_3MB shifts to lower temperatures, which may be attributed to polymer degradation caused by the additional thermal processing step involved in the masterbatch preparation. This behavior aligns with previous reports showing thermal degradation and reduced molecular weight in reprocessed PLA [30]. The significant shift in PLA_3MB, therefore, suggests considerable thermal degradation at higher Gel-mod loadings when processed via the masterbatch route.

### 3.3. Processability of PLA Samples from MFI Measurements

**Figure 7:**
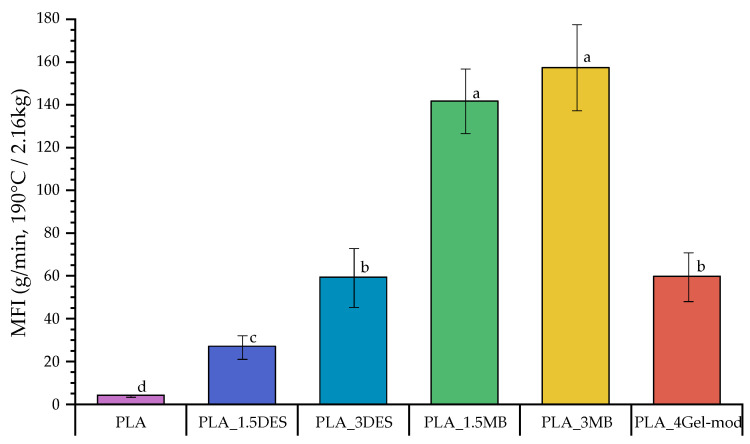
Melt flow index of PLA samples at 190 °C, using a 2.16 kg load. Means with different letters are significantly different (ANOVA + Tukey’s post hoc test, *p* < 0.05).

Melt flow index (MFI) analysis is an important parameter for assessing its processability under flow conditions. The MFI results for the PLA-based formulations are presented in Figure 7. Neat PLA exhibits a low MFI value (4.2 g/10 min), consistent with the high melt viscosity typically associated with extrusion-grade PLA. As demonstrated previously by DSC analysis, incorporating DES significantly increases polymer chain mobility. This is reflected in the substantial increase in MFI observed for PLA_1.5DES and PLA_3DES (27 and 60 g/10 min, respectively), confirming the plasticizing and lubricating effects of DES molecules such as glycerol, which reduce intermolecular interactions and facilitate melt flow [31,32]. An even more pronounced effect is observed for formulations containing the gelatin-based masterbatch. PLA_1.5MB and PLA_3MB exhibit dramatic increases in MFI, exceeding 140 g/10 min, representing more than an order of magnitude increase relative to neat PLA. This enhancement surpasses that observed for the DES-only systems, suggesting that the incorporation of Gel-mod through the masterbatch route modifies melt behavior not solely through plasticization but also through polymer chain degradation, which is consistent with a decrease in molecular weight. In contrast, the direct incorporation of Gel-mod into the PLA matrix (PLA_4Gel-mod) results in a more moderate MFI increase, comparable to PLA_3MB. This indicates that single-step incorporation does not induce significant thermal degradation of PLA, unlike the masterbatch approach involving an additional thermal cycle. These results highlight the critical importance of minimizing processing steps in polyester-based systems due to their inherent susceptibility to thermally induced chain scission [30].

### 3.4. Mechanical Properties of PLA Samples

The tensile test results (Table 4) show that neat PLA exhibits the characteristic behavior of a brittle polymer, presenting high tensile strength and elastic modulus but extremely low elongation at break. The incorporation of DES leads to a reduction in tensile strength, modulus, and elongation at break. Although DSC results indicated a decrease in Tg, suggesting a plasticizing effect, the simultaneous increase in crystallinity appears to dominate the mechanical response, promoting earlier crack initiation and reduced ductility. Accordingly, PLA_1.5DES and PLA_3DES display lower modulus and tensile strength relative to neat PLA due to plasticization, whereas the further reduction in elongation at break at higher DES content correlates with the increased crystalline fraction, which restricts chain mobility [33]. For the formulations incorporating Gel-mod through the masterbatch route, a more pronounced reduction in all mechanical properties is observed, particularly for PLA_3MB. This deterioration is likely associated with polymer chain scission induced by the additional thermal processing cycle, resulting in decreased molecular weight and, consequently, reduced strength and ductility. Despite containing a higher fraction of Gel-mod, PLA_4Gel-mod exhibits mechanical performance comparable to or superior to PLA_1.5MB, indicating that the direct incorporation method mitigates thermal degradation effects. The PLA_4Gel-mod formulation, therefore, provides a more favorable balance between stiffness and strength, although it is still inferior to neat PLA due to the combined effects of increased crystallinity and partial chain degradation. These mechanical trends are also consistent with the intrinsic incompatibility between PLA and gelatin (hydrophobic and hydrophilic natures, respectively), which hinders interfacial adhesion and contributes to the observed decrease in mechanical performance, as discussed in the literature [34].

### 3.5. Surface and Barrier Properties

#### 3.5.1. Contact Angle (CA)

**Figure 8:**
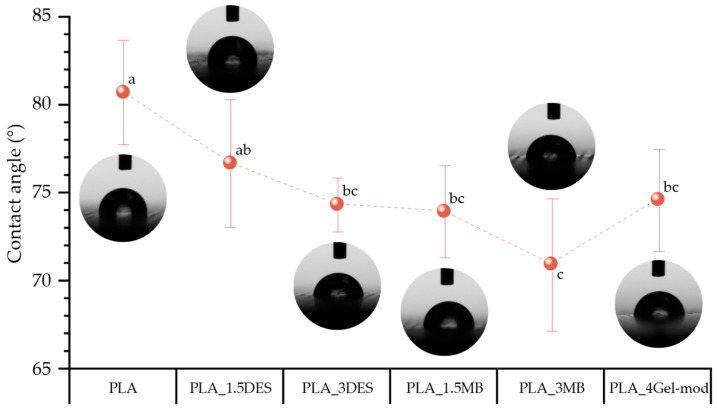
Contact angles of the produced films. Means with different letters are significantly different (ANOVA + Tukey’s post hoc test, *p* < 0.05).

The evaluation of static water contact angles reflects the ability of a material to interact with and spread a liquid droplet across its surface, with lower contact angle values indicating higher wettability. The average contact angle values, together with representative images of water droplets at 0 s after deposition, are presented in Figure 8. A progressive decrease in contact angle with the incorporation of DES and Gel-mod can be noticed, decreasing from 80.7° for neat PLA to values as low as 70.9°. This reduction indicates enhanced wetting of distilled water on the film surface. Moreover, increasing concentration from 1.5 to 3 (*w*/*w*) (DES- or MB-based formulations) results in a more pronounced reduction in contact angle, further confirming the increased hydrophilicity. Although the PLA_4Gel-mod film exhibits a slightly higher contact angle (74.6°) than PLA_3MB (70.9°), the difference may not be statistically significant within the experimental variability. Overall, the observed reduction in contact angle for all modified formulations is consistent with the introduction of hydrophilic DES and Gel-mod components, which likely promote the formation of hydrogen-bonding interactions and orient polar functional groups toward the film surface, thereby enhancing affinity with water molecules [35,36].

#### 3.5.2. WVTR Analysis of PLA Samples

**Figure 9:**
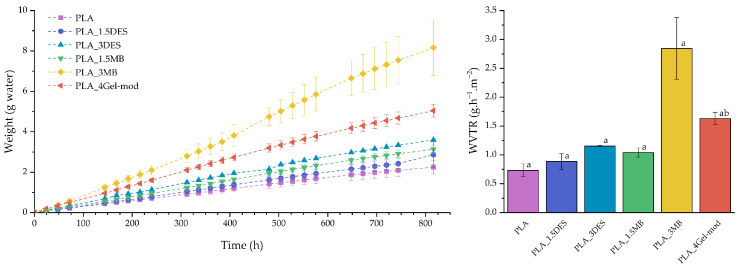
Water vapor transmission over time (**left**) and WVTR (**right**) of all the material films. Means with different letters are significantly different (ANOVA + Tukey’s post hoc test, *p* < 0.05).

WVTR analysis was conducted to evaluate the impact of DES and Gel-mod, at different incorporation levels, on the water vapor barrier performance of PLA-based films. The profiles of water mass uptake (Figure 9) exhibit nonlinear behavior for all formulations. An overall increase in WVTR was observed for the modified PLA films compared with neat PLA, indicating a reduction in barrier efficiency due to enhanced hydrophilicity of the incorporated components (DES, HEMA, and gelatin), which is consistent with the trends identified in the contact angle measurements. Notably, PLA_3MB displays a markedly higher WVTR, which can be partially attributed to its lower film thickness relative to the other formulations, as permeability is inversely proportional to thickness. The incorporation of 1.5% DES or 1.5% Gel-mod results in WVTR values of 0.84 and 0.94 g·h^−1^·m^−2^, respectively, which are not significantly different from each other. However, increasing DES loading, doubling the masterbatch content, or incorporating 4% Gel-mod substantially increased WVTR, with values of 1.05, 2.68, and 1.48 g·h^−1^·m^−2^ for PLA_3DES, PLA_3MB, and PLA_4Gel-mod, respectively. These findings indicate that the incorporation of modified gelatin containing HEMA and DES reduces the barrier to water vapor diffusion relative to neat PLA, likely due to the increased presence of polar hydrophilic groups and associated microstructural pathways facilitating moisture transport.

### 3.6. Compostability Acessment of PLA Samples

**Figure 10:**
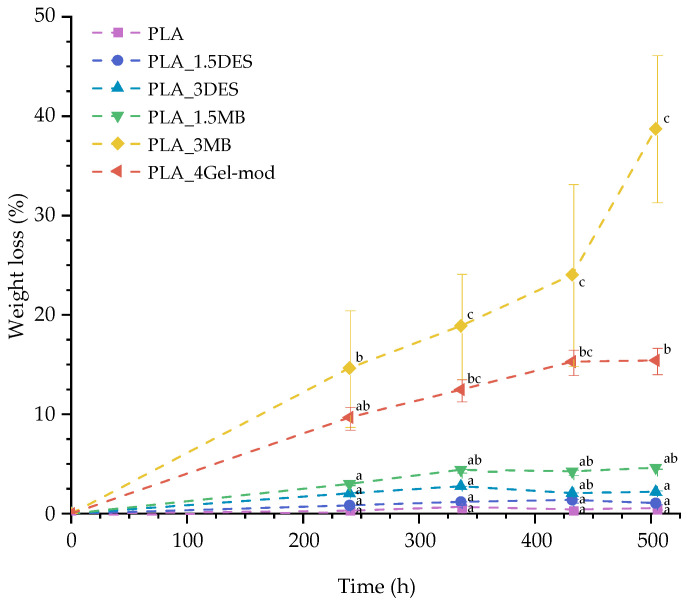
Evolution of weight loss of PLA samples under compostable conditions, at 40 °C. Means with different letters are significantly different (ANOVA + Tukey’s post hoc test, *p* < 0.05).

A major advantage of biopolymer-based films for food packaging is their ability to undergo controlled biodegradation under composting conditions. As indicated by the contact angle measurements, the developed films exhibit increased surface hydrophilicity relative to neat PLA; therefore, an enhancement in compostability was anticipated for formulations containing DES and Gel-mod. Composting tests were performed on neat PLA and the modified compositions (PLA_1.5DES, PLA_3DES, PLA_1.5MB, PLA_3MB, and PLA_4Gel-mod) over a 21-day period, with weight loss monitored at 5-day intervals, as in Figure 10.

Two distinct degradation profiles were identified. The first group, including PLA, PLA_1.5DES, PLA_3DES, and PLA_1.5MB, exhibited a nearly linear weight-loss trend, reaching approximately 6% weight reduction after 320 h. Within this group, PLA_1.5MB shows a slightly higher weight loss, possibly due to its higher hydrophilicity. In contrast, the formulations with higher Gel-mod content (PLA_3MB and PLA_4Gel-mod) display markedly different behavior: although both films initially follow a linear trend up to ~240 h, PLA_3MB subsequently presents an exponential increase in weight loss, reaching nearly 40% degradation by the end of the test period.

The pronounced biodegradation of PLA_3MB may be attributed to its reduced film thickness (0.12 mm) and enhanced hydrophilicity, which promote moisture uptake and accelerate microbial activity and hydrolytic chain scission. These findings are consistent with previous reports indicating that the incorporation of gelatin can effectively modulate the biodegradation rate of PLA-based materials, acting as a green strategy to enhance end-of-life performance [37].

## 4. General Discussion

This study demonstrates that the incorporation of modified gelatin (Gel-mod) and deep eutectic solvent (DES) into PLA significantly affects the physicochemical, mechanical, thermal, and processability properties of the PLA. The combined results indicate that both additives affect the polymer matrix through mechanisms involving plasticization, crystallinity modification, interfacial interaction, and increased hydrophilicity.

Morphological analysis by SEM revealed clear differences in fracture behavior among the formulations, indicating that DES incorporation produces a more uniform microstructure, which is consistent with previous studies reporting enhanced polymer fluidity and film homogeneity in DES-plasticized films [11]. In contrast, gelatin addition (particularly via the masterbatch route) resulted in heterogeneous morphology with visible interfacial discontinuities, as already observed in Rodrigues et al. and Valvés et al. [26,38]. The PLA_4Gel-mod formulation exhibited improved dispersion of Gel-mod and reduced phase visibility, suggesting enhanced compatibility when gelatin is introduced directly without any additional thermal cycle.

Thermal analysis supported these observations. TGA results confirmed multiple degradation events corresponding to the thermal behavior of the Gel-mod and DES. Thus, these results are in accordance with the literature, since it was expected that the chemical nature of CHCl/Gly would affect the gelatin stability [13,39]. Gel-mod-containing films showed slightly reduced thermal stability relative to neat PLA, while DES-only systems maintained comparable degradation temperatures. DSC results are in agreement with reported literature, revealing that DES acts as an effective plasticizer, reducing Tg and cold crystallization temperatures and enhancing chain mobility [11,40]. Along with this, crystallinity increased markedly, up to 48%, indicating that DES also functions as a nucleating agent. The masterbatch formulations exhibited shifts in melting temperature attributed to additional thermal processing and associated chain scission, corroborating the elevated MFI values and supporting degradation-induced viscosity reduction mechanisms.

The mechanical behavior of the films was strongly correlated with these structural changes. DES plasticization reduced stiffness and strength, and masterbatch systems displayed pronounced deterioration due to thermal degradation and reduced molecular weight. In contrast, direct incorporation (PLA_4Gel-mod) provided a more favorable balance between stiffness and ductility, underscoring the relevance of processing route optimization in polyester systems. Weerasinghe et al. also demonstrated that DES acts as a plasticizer, increasing elongation/toughness and improving ductility while preserving stiffness [11].

Surface wettability and vapor barrier properties proved highly sensitive to hydrophilic additive content. Contact angle measurements confirmed increasing hydrophilicity with Gel-mod and DES incorporation, which correlated with greater water vapor permeability. Marco-Velasco et al. and Poulain et al. reported that the presence of both gelatin and DES increases surface hydrophilicity and water vapor permeability in polymeric films due to their higher affinity for water [12,41]. PLA_3MB exhibited the highest WVTR, consistent with its hydrophilic surface, compromised morphology, and reduced thickness. These findings confirm that polymer chain orientation, additive distribution, and surface polarity collectively determine permeability behavior.

Finally, compostability tests demonstrated accelerated biodegradation for formulations with higher Gel-mod content, with PLA_3MB achieving nearly 40% mass loss after 320 h. This enhancement was attributed to increased wettability and reduced thickness, facilitating hydrolytic and microbial activity. These results align with previous studies reported in the literature, which reveal that proteinaceous additives enhance the biodegradation rate of PLA by promoting water penetration and enzymatic accessibility [26,37,42].

Overall, the results demonstrate that the synergistic incorporation of DES and gelatin-mod enables tunable modification of PLA film performance, offering a viable strategy to tailor processability, mechanical behavior, barrier properties, and biodegradability for food packaging applications. These findings emphasize the importance of processing methodology, additive distribution, and molecular interactions in optimizing biopolymer composite design. Future work should investigate the incorporation of active compounds and performance evaluation under real food storage conditions to further develop these films as advanced materials for sustainable active packaging.

## 5. Conclusions

This study demonstrates the feasibility of tailoring the properties of PLA-based films for sustainable food packaging applications through the incorporation of modified gelatin (Gel-mod) and deep eutectic solvents (DES). The results show that DES effectively plasticizes the PLA matrix, enhancing molecular mobility, increasing crystallinity, and improving melt flow behavior without causing substantial reductions in thermal stability.

The incorporation of Gel-mod further modified the polymer matrix, particularly affecting crystalline morphology, surface hydrophilicity, and biodegradation behavior. However, the extent of these effects was strongly dependent on the processing route. The masterbatch method, which introduces an additional thermal cycle, led to significant PLA chain degradation and, consequently, reduced mechanical performance. In contrast, direct incorporation of Gel-mod (PLA_4Gel-mod) yielded films with more homogeneous morphology, improved mechanical stability, and reduced degradation, underscoring the importance of minimizing thermal exposure during processing.

Surface wettability and barrier properties analyses revealed increased hydrophilicity and reduced water vapor barrier performance in all modified films, consistent with the introduction of polar groups from DES, HEMA, and gelatin. These modifications correlated with accelerated biodegradation, particularly in formulations with higher Gel-mod content, demonstrating the potential of gelatin as a natural pro-degradation agent to fine-tune end-of-life performance.

Overall, this study demonstrates that the synergistic use of DES and Gel-mod enables tailored modification of PLA properties, offering a promising strategy for developing compostable films with customizable mechanical, barrier, and degradation characteristics for food packaging applications. Future studies should explore the incorporation of active compounds and evaluate film behavior under real storage conditions to advance their potential use in active and intelligent packaging systems.

## Figures and Tables

**Table 1:** Composition of Gel-mod.

Reagents	ChCl (wt.%)	Glycerol (wt.%)	Gelatin (wt.%)	HEMA (wt.%)
Gel-mod	21.5	42.5	21	15

**Table 2:** Composition of the PLA composites.

Compositions	PLA (%)	DES-HEMA (%)	Gel-Mod (%)	Ratio Gelatine/PLA (%)	Ratio DES-HEMA/PLA (%)	t ^1^ (mm)
PLA	100.0	-	-	-	-	0.17 ± 0.02
PLA_1.5DES	94.3	5.7	-	-	6	0.17 ± 0.02
PLA_3DES	88.4	11.6	-	-	13	0.15 ± 0.02
PLA_1.5MB	92.9	-	7.1	1.6	6	0.15 ± 0.01
PLA_3MB	85.7	-	14.3	3.5	13	0.12 ± 0.01
PLA_4Gel-mod	81.0	-	19.0	4.9	19	0.16 ± 0.01

^1^ Note: This is the final average thickness of the films obtained by compression molding.

**Table 3:** Thermal parameters obtained from the TGA and DSC analysis.

Composition	Tg (°C)	Tc (°C)	Tm (°C)	∆Hc (J/g)	∆Hm (J/g)	Xc *(*%*)*	T_peak_ (°C)
PLA	58	120	148/155	−8.8	10.0	11	317
Gel-mod	-	-	-	-	-	-	292
PLA_1.5DES	55	108	143/152	−35.3	37.8	43	320
PLA_3DES	54	109	142/152	−40.2	39.9	48	320
PLA_1.5MB	55	113	142/150	−39.8	39.3	45	306
PLA_3MB	53	109	139/148	−30.3	29.3	37	318
PLA_4Gel-mod	54	117	143/151	−31.6	31.7	42	325

Note: T_peak_ is the highest degradation temperature peak; Tg is the glass transition temperature; Tc and Tm are the cold crystallization and the melting temperature, respectively; ∆Hc and ∆Hm are the crystallization and melting enthalpy, respectively, and X_c_ is the degree of crystallinity of the PLA fraction. The standard deviation is below 5% of the mean value.

**Table 4:** Mechanical properties from tensile tests of PLA samples. Means with different letters are significantly different (ANOVA + Tukey’s post hoc test, *p* < 0.05).

Composition	Elastic Modulus (MPa)	Tensile Strength (MPa)	Elongation at Break (%)
PLA	1733 ± 209 ^a^	45.2 ± 5.2 ^a^	4.6 ± 0.7 ^a^
PLA_1.5DES	1693 ± 128 ^a^	36.3 ± 2.3 ^b^	3.9 ± 0.9 ^ab^
PLA_3DES	1560 ± 261 ^a^	28.4 ± 1.6 ^d^	3.6 ± 0.4 ^ab^
PLA_1.5MB	1536 ± 152 ^a^	18.2 ± 2.6 ^d^	1.4 ± 0.1 ^cd^
PLA_3MB	779 ± 449 ^b^	2.3 ± 0.7 ^e^	0.3 ± 0.1 ^d^
PLA_4Gel-mod	1333 ± 25 ^a^	19.3 ± 1.3 ^c^	2.3 ± 0.5 ^bc^

## Data Availability

The data presented in this study is available in the article.
